# A methodologic survey on use of the GRADE approach in evidence syntheses published in high-impact factor urology and nephrology journals

**DOI:** 10.1186/s12874-022-01701-x

**Published:** 2022-08-10

**Authors:** Shuang Zhang, Qi-Jun Wu, Shu-Xin Liu

**Affiliations:** 1 Department of Nephrology, Dalian Municipal Central Hospital, No.826, Xinan Road, Dalian, Liaoning 116033 China; 2 Dalian Key Laboratory of Intelligent Blood Purification, Dalian Municipal Central Hospital, No.826, Xinan Road, Dalian, Liaoning 116033 China; 3 Department of Clinical Epidemiology, Shengjing Hospital of China Medical University, Shenyang, China

**Keywords:** GRADE, Non-randomized studies, Randomized control trials, Systematic review, Urology and nephrology

## Abstract

**Background:**

To identify and describe the use of the Grading of Recommendations, Assessment, Development and Evaluation (GRADE) approach for rating the certainty of systematic reviews (SRs) evidence published in urology and nephrology journals.

**Methods:**

SRs that were published in the top ten "urology and nephrology" journals with the highest impact factor according to the 2020 Journal Citation Reports (covering 2016–2020) were systematically searched and evaluated using the GRADE approach.

**Results:**

A total of 445 SRs were researched. Sixty SRs of randomized control trials (RCTs) and/or non-randomized studies (NRSs) were evaluated using the GRADE approach. Forty-nine SRs (11%) rated the outcome-specific certainty of evidence (*n* = 29 in 2019–2020). We identified 811 certainty of evidence outcome ratings (*n* = 544 RCT ratings) as follows: very low (33.0%); low (32.1%); moderate (24.5%); and high (10.4%). Very low and high certainty of evidence ratings accounted for 55.0% and 0.4% of ratings in SRs of NRSs compared to 23.0% and 15.3% in SRs of RCTs. The certainty of evidence for RCTs and NRSs was downgraded most often for risk of bias and imprecision.

**Conclusions:**

We recommend increased emphasis on acceptance of the GRADE approach, as well as optimal use of the GRADE approach, in the synthesis of urinary tract evidence.

**Supplementary Information:**

The online version contains supplementary material available at 10.1186/s12874-022-01701-x.

## Introduction

The Grading of Recommendations, Assessment, Development and Evaluation (GRADE) approach is a system for rating the quality of a body of evidence in systematic reviews (SRs) and other evidence syntheses, such as health technology assessments, and guidelines and grading recommendations in health care [1]. GRADE approach offers a transparent and structured process for developing and presenting evidence summaries and for carrying out the steps involved in developing recommendations. It can be used to develop clinical practice guidelines and other health care recommendations (e.g. in public health, health policy and systems and coverage decisions), and is becoming increasingly popular among guideline developers and systematic reviewers [1]. Up to date, more than 110 organizations from 19 countries around the world have endorsed GRADE approach, including the World Health Organization, the Kidney Disease: Improving Global Outcomes (KDIGO) guidelines, the World Allergy Organization, and the World Society of the Abdominal Compartment Syndrome and the Cochrane Collaboration [1]. In addition, it is also becoming an international standard for judging the evidence in SRs and clinical guidelines [2]. GRADE approach differs from other appraisal tools for three reasons: (i) because it separates quality of evidence and strength of recommendation, (ii) the quality of evidence is assessed for each outcome, and (iii) observational studies can be ‘upgraded’ if they meet certain criteria [3]. Thus, appropriate application of the GRADE approach can provide quality of evidence and strength of recommendations that is explicit, comprehensive, transparent, and pragmatic.

Systematic reviews (SRs) attempt to identify, select, synthesize, and appraise all high-quality research evidence relevant to a well-honed question. There have been many studies based on SRs, such as the GBD database [4], the World Cancer Research Fund/American Institute for Cancer Research report [5], as well as dietary guidelines [6]. As far as we know, untreated urological conditions are the major burden of patients all over the world. So far, many studies have summarized the factors affecting the urinary system and the prognosis of the urinary system disease using SRs.

However, no studies to date have assessed how SRs in urology and nephrology journals have used the GRADE approach to evaluate the certainty (or quality) of evidence. Therefore, the purpose of this study was to identify and describe all relevant SRs that use the GRADE approach to evaluate the outcome-specific certainty of evidence published from 2016–2020 in the top 10 urology and nephrology journals with the highest impact factor according to the 2020 Journal Citation Reports (JCR), and to summarize and present the GRADE-specific information, such as the outcomes rated, the number of primary studies, the exposure category, the use of summary of findings tables, the total number of down- and up-grading domains, while also considering the study design (SRs of randomized-controlled trials RCTs vs. non-randomized studies NRSs).

## Methods

### Search strategy

SRs published in the top 10 urology and nephrology journals with the highest impact factor according to the 2020 JCR between 1 January 2016 and 31December 2020 were identified by searching in the PubMed database (Supplementary Appendix 1).

### Selection of documents

The SRs were included when the following criteria were met: (1) SRs utilizing the GRADE approach to assess the certainty of evidence. We excluded the following SRs: (1) a modified version of the GRADE approach was applied to assess the certainty of evidence; and (2) failure to provide details of the GRADE approach evaluation process and results.

First, two reviewers (SZ, S-X L.) independently screened identified articles by title and abstract. Only specific irrelevant articles were excluded at this stage. Second, we independently obtained and checked all potentially relevant articles for final inclusion by two reviewers (SZ, S-X L.). Selection disagreements were resolved by discussion or consultation with a third author (Q-J W.).

### Data extraction

We extracted the following information for all identified SRs: year of publication; journal name; number of primary studies included; type of studies included RCTs vs. NRSs (including, non-randomized intervention, case–control, cohort, and cross-sectional studies) vs. combination RCTs/NRSs; number of participants; description of intervention(s)/exposure(s); number and types of outcome(s) and comparison(s) rated; category of certainty of evidence ratings (high, moderate, low, or very low); meta-analysis conducted (yes vs. no); summary of findings table reported (yes vs. no); number of down- and/or up-grading (count of the respective downgrading/upgrading domain used at the outcome level); and reasons for down- and/or up-grading. The one reviewer (SZ) extracted the study characteristics of the SRs, then one reviewer cross-checked all data (S-X L). We extracted all downgrading factors listed in the SRs of RCTs and NRSs according to the study design. There were five SRs with pooled RCTs and NRSs in which the combined evidence was rated [7–11].

We referred to minimum criteria proposed by GRADE working group when rating the certainty of evidence in this GRADE methodologic survey, which refers to the evidence that was assessed and the methods that were used to identify and appraise that evidence were not be clearly described for SRs that were assessed in our study.

## Results

### Search results and sample

**Fig. 1:**
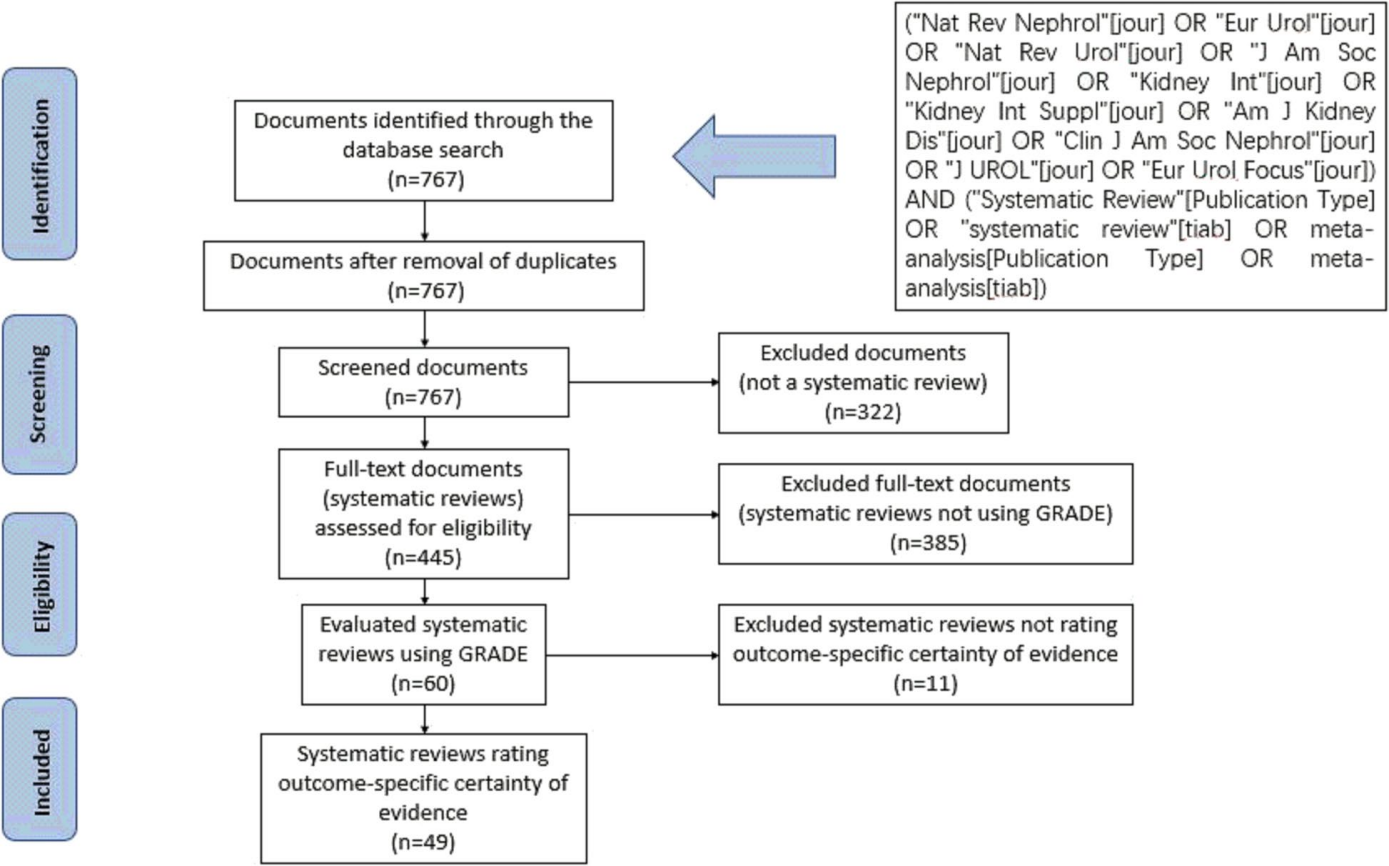
Flow diagram showing study selection process

A flow diagram of the literature search is shown in Fig. 1. The initial search yielded 767 entries, of which 322 were excluded that were not SRs. Of the 445 SRs identified, 60 met the eligibility criteria [7–66]. Out of the 60 studies, 11 studies [56–66] (18.33%) did not report on outcome-specific rating, which refers to certainty of evidence of individual studies was assessed or overall certainty of the body of evidence was rated (Supplementary Table S1). Therefore, a total of 49 SRs (81.67%) that rated the outcome-specific certainty of evidence were included in our methodologic survey.

The distribution of the 49 SRs according to the journal and published year are presented in Table 1. The greatest and least number of SRs published in the top 10 urology and nephrology journals was in 2016 (*n* = 115) and 2018 (*n* = 67), respectively. In addition, there was an increase in the proportion of SRs that rated the certainty of evidence using the GRADE approach among all SRs published in 2019 and 2020 (17.7% and 14.4%, respectively) compared to 2016, 2017, and 2018 (6.1%, 10.0%, and 7.5%, respectively). Four journals (*Journal of Urology*, *European Urology*, *Clinical Journal of the American Society of Nephrology*, and *American Journal of Kidney Diseases*) accounted for 86% of all SRs, whereas 3 journals (*Kidney International Supplements*, *Nature Reviews Urology*, *Nature Reviews Nephrology*) did not publish SRs using the GRADE approach.

**Table 1:** The distribution of systematic reviews (*n* = 49) that rated the outcome-specific certainty of evidence with GRADE by year and journal

	**Number of SRs published, n**	**Number of SRs rating the outcome specific certainty of evidence with GRADE, n (% of SRs published)**	**Impact factor**	**Journal category**
**Total, n**	445	49 (11.0%)	-	-
2016	115	7 (6.1%)	-	-
2017	80	8 (10.0%)	-	-
2018	67	5 (7.5%)	-	-
2019	79	14 (17.7%)	-	-
2020	104	15 (14.4%)	-	-
**Journal**				
Journal of Urology	100	13 (13.0%)	7.45	Urology & Nephrology
European Urology	128	12 (9.4%)	20.096	Urology & Nephrology
Clinical Journal of The American Society of Nephrology	38	10 (26.3%)	8.237	Urology & Nephrology
American Journal of Kidney Diseases	45	7 (15.6%)	8.86	Urology & Nephrology
European Urology Focus	110	4 (3.6%)	5.996	Urology & Nephrology
Journal of The American Society of Nephrology	14	2 (14.3%)	10.121	Urology & Nephrology
Kidney International	10	1 (10.0%)	10.612	Urology & Nephrology
Kidney International Supplements	0	0	10.545	Urology & Nephrology
Nature Reviews Urology	0	0	14.432	Urology & Nephrology
Nature Reviews Nephrology	0	0	28.314	Urology & Nephrology

*SRs* Systematic reviews

### Characteristics of the included SRs

SRs analyzing evidence from only RCTs (*n* = 20) [12, 14, 15, 18, 20, 22, 24, 26, 30, 32–37, 40, 41, 43, 45, 46], only NRSs (*n* = 15) [19, 21, 23, 31, 39, 42, 44, 47–54], or RCTs and NRSs (*n* = 14) [7–11, 13, 16, 17, 25, 27–29, 38, 55] were included into this methodological study. The main features of these 49 eligible SRs are summarized in Supplementary Table S2. Among the 14 SRs that combined RCTs and NRSs, 5 rated the outcome-specific certainty of evidence derived from RCTs and NRSs separately (in separate rows within a single Summary of Findings table or in separate Summary of Findings tables) [8, 17, 25, 27, 28], 5 pooled RCTs and NRSs and rated the combined evidence (in the same rows within a single Summary of Findings table) [7–11], and 6 only rated the NRSs [9–11, 13, 29, 38].

Forty-six SRs (94%) conducted at least one meta-analysis [7–37, 39–52, 55], and 39 SRs (80%) presented the findings in a summary of findings table [8–13, 15, 17–23, 25–34, 36–38, 40–43, 45–51, 53]. The median number of primary studies included in the SRs was 19 (range = 5 to 104). The median number of participants included in the SRs based on RCTs was 4253 (range = 484 to 16,990), 14,550 (range = 1394 to 2,791,732) in the SRs based on NRSs, and 2958 (range = 708 to 8163) in the SRs based on RCTs and NRSs. Additionally, we classified the interventions and exposures in the SRs as clinical therapies (*n* = 22), drugs (*n* = 14), specific clinical diseases (*n* = 6), lifestyle factors (*n* = 3), supplements (*n* = 2), clinical approaches (*n* = 1), and clinical care (*n* = 1) (Table 2).

**Table 2:** Summary of the systematic reviews characteristics

	**Total SRs (*n* = 49)**	**SRs of RCTs** **(*n* = 20)**	**SRs of NRSs** **(*n* = 15)**	**SRs of RCTs/NRSs** **(*n* = 14)**
Number of primary studies, median (range)	19 (5–104)	22 (5–90)	20 (7–104)	19 (5–61)
Number of participants, median (range)	4222 (484–2,791,732)	4253 (484–16,990)	14,550 (1394–2,791,732)	2958 (708–8163)
Summary of findings table, n	39	17	11	11
Meta-analysis conducted, n	46	20	13	13
Outcomes rated in a SR, median (range)	8 (1–184)	5 (1–249)	5 (1–78)	5 (1–68)
Category				
Clinical therapy, n	22	8	7	7
Drug, n	14	10	1	3
Specific clinical disease, n	6	0	4	2
Lifestyle factors, n	3	0	3	0
Supplements, n	2	1	0	1
Clinical approach, n	1	0	0	1
Clinical care, n	1	1	0	0

*NRSs* Nonrandomized studies, *RCTs* Randomized controlled trials, *SRs* Systematic reviews

### Certainty of evidence ratings

The median number of outcomes rated in a SR was 8 (range = 1 to 184) (Table 2). There were 811 individual outcome ratings (544 for RCTs and 256 for NRSs) The quality of evidence was assessed in 4 categories, as follows: very low (33%); low (32.1%); moderate (24.5%); and high (10.4%). Outcomes were rated in 23.0%, 34.0%, 27.7%, and 15.3% as a very low, low, moderate, and high certainty of evidence among SRs based on RCTs, respectively. The certainty of evidence was rated as very low (55.0%), low (27.0%), moderate (17.6%), and high (0.4%) among SRs based on NRSs. Among SRs that combined NRSs and RCTs, 11 outcomes were rated; the certainty of evidence was rated as very low (18.2%), low (54.5%), and moderate (27.3%) (Table 3).

**Table 3:** Details on the certainty of evidence ratings including down- and upgrading’s of the evidence according to study design

	**Total (%)**	**RCTs (%)**	**NRSs (%)**	**RCTs/NRSs (%)**
**The rating of the certainty per outcome, n**	**811**	**544**	**256**	**11 (5 studies)**
High, n (%)	84 (10.4)	83 (15.3)	1 (0.4)	0
Moderate, n (%)	199 (24.5)	151 (27.7)	45 (17.6)	3 (27.3)
Low, n (%)	260 (32.1)	185 (34.0)	69 (27.0)	6 (54.5)
Very low, n (%)	268 (33.0)	125 (23.0)	141 (55.0)	2 (18.2)
**Total number of downgrading domains, n**	**1012**	**667**	**339**	**6**
Risk of bias, n (%)	528 (52.2)	358 (53.8)	167 (49.3)	3 (50.0)
Imprecision, n (%)	278 (27.5)	207 (31.0)	71 (21.0)	0
Inconsistency, n (%)	146 (14.4)	84 (12.6)	60 (17.7)	2 (33.3)
Indirectness, n (%)	55 (5.4)	17 (2.5)	37 (10.8)	1 (16.7)
Publication bias, n (%)	5 (0.5)	1 (0.1)	4 (1.2)	0
**Upgradings, n**	**21**	**0**	**19**	**2**
Large effect, n (%)	3 (14.3)	0	1 (5.3)	2 (100.0)
Dose–response, n (%)	10 (47.6)	0	10 (52.6)	0
Plausible confounding, n (%)	8 (38.1)	0	8 (42.1)	0
**Frequency of the rating domains**				
**Mean frequency, n of Downgradings domains, n / The rating of the certainty per outcome, n**	**1.25** **1012/811**	**1.23** **667/544**	**1.32** **339/256**	**0.55** **6/11**
Risk of bias, n (% of outcomes downgraded)	528 (65.1)	358 (65.8)	167 (65.2)	3 (27.3)
Imprecision, n (% of outcomes downgraded)	278 (34.3)	207 (38.0)	71 (27.7)	0
Inconsistency, n (% of outcomes downgraded)	146 (18.0)	84 (15.4)	60 (23.4)	2 (18.2)
Indirectness, n (% of outcomes downgraded)	55 (6.8)	17 (3.1)	37 (14.5)	1 (9.1)
Publication bias, n (% of outcomes downgraded)	5 (0.6)	1 (0.2)	4 (1.6)	0
** Mean frequency, n of Upgrading domains, n / The rating of the certainty per outcome, n**	**0.03** **21/811**	**0.00** **0/544**	**0.07** **19/256**	**0.18** **2/11**
Large effect, n (% of outcomes upgraded)	3 (0.4)	0	1 (0.4)	2 (18.2)
Dose–response, n (% of outcomes upgraded)	10 (1.2)	0	10 (3.9)	0
Plausible confounding, n (% of outcomes upgraded)	8 (1.0)	0	8 (3.1)	0

*RCTs* Randomized controlled trials, *SRs* Systematic reviews

### Up- and down-grading domains

A total of 1012 instances of downgrading were identified due to a risk of bias (RoB; 52.2%), imprecision (27.5%), inconsistency (14.4%), indirectness (5.4%), and publication bias (0.5%). According to the authors of those SRs, rating down for publication bias was as a result of asymmetry of the funnel plot or it was strongly suspected the study design (patient reports published in toxicology report very severe poisoning either with or without impressive recovery with treatments attempted) [43, 53]. Twenty-one upgrades of the certainty of evidence were due to a large effect (14.3%), dose–response (47.6%), and plausible confounding (38.1%). The authors of those SRs considered that the all plausible residual confounding in the included studies would reduce the demonstrated effect [21, 29, 53]. Downgrading for RoB was more common in SRs of RCTs (53.8%) than SRs of NRSs (49.3%), whereas downgrading for publication bias was more common in SRs of NRSs (1.2%) than SRs of RCTs (0.1%). Additionally, upgrading for large effect, dose–response, and plausible confounding were all in SRs of NRSs (Table 3).

We calculated 1.2 mean downgrades per outcome in SRs of RCTs and 1.3 mean downgrades per outcome in SRs of NRSs. The downgrading frequency (the number of downgrades per number of rated outcomes) among SRs of RCTs for the RoB (65.8%) and imprecision (38.0%) domains was greater than the SRs of NRSs. In contrast, the downgrading frequency among SRs of NRSs for the inconsistency (23.4%), indirectness (14.5%), and publications bias (1.6%) was greater than the SRs of RCTs (Table 3). In addition, 7% of outcomes rated in SRs of NRSs were upgraded; 3.9% of outcomes were upgraded for dose–response, 3.1% for plausible confounding, and 0.4% for a large effect. The reasons for the downgrade or upgrade of the certainty of evidence for outcomes is shown in Supplementary Table S3.

## Discussion

### Summary of findings

This is the first study to evaluate the application of the GRADE approach in SRs published in the top 10 urology and nephrology journals. In general, there are relatively few urology and nephrology SRs using GRADE approach to rate the certainty of evidence, but an increasing trend in the level of implementation was noted in the past 2 years [9, 11, 17, 19–29, 31, 33, 40–43, 45–51, 53, 55]. We identified 49 SRs that rated the outcome-specific certainty of evidence. Overall, a low and very low certainty of evidence accounted for 32.1% and 33% of the individual outcomes, respectively. In addition, the certainty of evidence was downgraded most of for RoB and imprecision among SRs of RCTs and NRSs.

### Strengths and limitations

There were several strengths in our study. First, we have searched all published SRs that utilized the GRADE approach among the top 10 urology and nephrology journals from 2016–2020. A wide range of relevant information was extracted, such as the classification of outcomes assessment, and the number and reasons for down- and up-grading domains. Second, this is the first study to summarize and present the GRADE-specific information on SRs published in urology and nephrology journals, which can provide essential information for follow-up research. There were also some limitations in our study. First, our results are limited due to the risk of selection bias. We only included the top 10 urology and nephrology journals with the highest impact factors within 5 years (*n* = 60). Therefore, we failed to obtain information from medical journals with lower impact factors. Nevertheless, the journals with the highest impact factor are persuasive, which can provide a basis for future clinical research. Second, because there was no clear and definite research protocol to follow, we adopted the PRISMA framework to report our findings. In fact, the PRISMA framework can provide comprehensive content and is often used in SRs and meta-analyses. Third, our study was based on a descriptive examination of the application of the GRADE approach in urology and nephrology SRs rather than to determine if the SR authors correctly followed guidance issued by the GRADE working group to rate the certainty of evidence. In the process of data extraction, we found that some authors did not follow minimum criteria of GRADE approach in the evaluation. Specifically, the certainty of evidence in some SRs was upgraded due to a low RoB, narrow confidence interval, a very low *P* value, and/or mild statistical heterogeneity rather than undergoing evaluation according to the GRADE approach domains for upgrading. Therefore, future research should have a priority for focusing on the optimal use of the GRADE approach, so that those applying the approach can have awareness brought to the main issues and difficulties faced when applying GRADE approach, to be able to correct them. Finally, this report does not address or assess any potential time trends. It is possible that the adoption of GRADE approach has increased and improved over time.

### Findings from other studies

The GRADE approach has been used in the fields of urology and nephrology during the recent 5 years. KDIGO Working Group formulated the scope of guidelines and graded evidence according to the GRADE approach, which now serves as the practice standard for KDIGO [67]. Similar to the findings in our study, the level of evidence was very low in most cases in the study conducted by Zare and colleagues [68]. The most frequent limitation involved indirectness due to the limited number of studies for each pair. In addition, the evidence quality was mainly downgraded due to the RoB and inconsistency. Moreover, presenting comprehensive evidence from RCTs and NRSs, as in our study, was similar to the Cuello-Garcia et al. study [69], which indicated that most respondents presented integrated data separately from RCTs and NRSs, either in a single summary of findings table or a standalone table. Compared to our study, these studies or guidelines merely indicated the use of the GRADE approach when rating the certainty of evidence or the preferences of experts when integrating RCTs and NRSs.

### Implications for broader research

Although the GRADE approach has been used for RCTs and NRSs in the fields of urology and nephrology, there are still some challenges in evaluating the certainty of evidence in NRSs with the GRADE approach. On the basis of the GRADE approach, the quality grade for an aggregate of RCTs would begin at the high entry level. A collection of observational studies would begin at the low entry level and evidence from other study designs, such as case–control studies, would begin at the very low level [70]. The GRADE approach, especially with respect to RoB assessment, is challenging and could lead to excessive downgrading. GRADE approach users may inappropriately double calculate the confounding and selection bias risk by downgrading the initial body of evidence to low, followed by further downgrading owing to unknown confounders in observational studies. In our study, in addition to the initial downgrading of two levels according to the study design, 53% of the SRs based on NRSs were further downgraded due to RoB.

The GRADE approach requires separate evaluations of RCTs or NRSs by any validated tool, but the specific method is not recommended because the selection of tools depends on the context [71]. With the emergence of tools that use the concept of target trials as a reference point, such as risk of bias in non-randomized intervention studies (ROBINS-I), the initial certainty of evidence is also considered high for bodies of evidence from NRSs [72, 73]. Several SRs have used the ROBINS-I tool in the field of nephrology thus far [74–76]. ROBINS-I can more systematically and accurately assess the RoB in NRSs to avoid extensive downgrading [73]. In general, the purpose of integrating rigorous RoB assessment into the GRADE approach, such as the application of the ROBINS-I tool in the SRs based on NRSs, is to improve the trustworthiness and credibility of the specific evidence [73]. To accurately rate the certainty of evidence, it is recommended to follow the current standards of RoB assessments, such as the ROBINS-I and the RoB tool by Cochrane [73, 77].

## Conclusion

The GRADE approach provides a system for rating quality of evidence and strength of recommendations that is explicit, comprehensive, transparent, and pragmatic. The GRADE approach is increasingly adopted by professional organizations worldwide. Our study demonstrated that the GRADE approach is not widely used (only 13.5% (60/445) of SRs reported using GRADE approach), within urology and nephrology SRs in top 10 journals. Thus, future research should focus on the optimal use of the GRADE approach by following the criteria proposed by the GRADE working group.

## Supplementary Information

**Supplementary file 1: Appendix 1.** Search strategy. **Table S1.** Overview of SRs that applied GRADE inappropriately a by rating the certainty of evidence for all studies or each individual study. **Table S2.** Summary of the study characteristics and the number and type of (un)rated outcomes of systematic reviews (n = 49) that rated the outcome-specific certainty of evidence with GRADE. **Table S3.** Overview of the GRADE domains of systematic reviews that rated the outcome-specific certainty of evidence.

## Data Availability

The datasets used and/or analyzed during the current study are available from the corresponding author upon reasonable request.

## Abbreviations

GRADE: Grading of recommendations, assessment, development and evaluation
GBD: Global burden of disease
JCR: Journal citation reports
KDIGO: Kidney disease: improving global outcomes
NRSs: Non-randomized studies
RCTs: Randomized control trials
RoB: Risk of bias
SRs: Systematic reviews
