# Supplementary material for: A methodologic survey on use of the GRADE approach in evidence syntheses published in high-impact factor urology and nephrology journals
Source: BMC Med Res Methodol. 2022 Aug 10;22:220. doi: 10.1186/s12874-022-01701-x (PMC9367121; doi:10.1186/s12874-022-01701-x)
Supplement: Supplementary file 1 — Appendix 1. Search strategy. Table S1. Overview of SRs that applied GRADE inappropriately a by rating the certainty of evidence for all studies or each individual study. Table S2. Summary of the study characteristics and the number and type of (un)rated outcomes of systematic reviews (n = 49) that rated the outcome-specific certainty of evidence with GRADE. Table S3. Overview of the GRADE domains of systematic reviews that rated the outcome-specific certainty of evidence. [file 12874_2022_1701_MOESM1_ESM.docx]

Supplementary Appendix 1. Search strategy

("Nat Rev Nephrol"[jour] OR "Eur Urol"[jour] OR "Nat Rev Urol"[jour] OR "J Am Soc Nephrol"[jour] OR "Kidney Int"[jour] OR "Kidney Int Suppl"[jour] OR "Am J Kidney Dis"[jour] OR "Clin J Am Soc Nephrol"[jour] OR "J UROL"[jour] OR "Eur Urol Focus"[jour]) AND ("Systematic Review"[Publication Type] OR "systematic review"[tiab] OR meta-analysis[Publication Type] OR meta-analysis[tiab])

Supplementary Table S1. Overview of SRs that applied GRADE inappropriately ^a^ by rating the certainty of evidence for all studies or each individual study

| **Author** | **Year** | **N, studies** | **Type of studies (n, particiapnts)** | **Intervention/Exposure** | **Application of GRADE** |
| --- | --- | --- | --- | --- | --- |
| Wagenaar ^1^ | 2017 | 18 | RCTs, NRSs (1686) | Minimally Invasive, Laparoscopic, and Robotic-assisted Techniques | Certainty of evidence of individual studies was assessed (not outcome specific) |
| Zaid ^2^ | 2016 | 8 | NRSs (2267) | CM | Certainty of evidence of individual studies was assessed (not outcome specific) |
| Han ^3^ | 2018 | 15 | NRSs (1163) | ^68^Ga-PSMA PET | Certainty of evidence of individual studies was assessed (not outcome specific) |
| Foroutan ^4^ | 2019 | 35 | NRSs (777,623) | Kidney Transplantation | Overall certainty of the body of evidence was rated (not outcome specific) |
| Campi ^5^ | 2018 | 22 | NRSs (565357) | Prostate cancer | Overall certainty of the body of evidence was rated (not outcome specific) |
| Kotecha ^6^ | 2020 | 8 | RCTs, NRSs (348) | Duloxetine use | Overall certainty of the body of evidence was rated (not outcome specific) |
| Reus ^7^ | 2018 | 12 | NRSs (886) | AUS | Certainty of evidence of individual studies was assessed (not outcome specific) |
| Cacciamani ^8^ | 2020 | 17 | RCTs, NRSs (13777) | Smoking status | Certainty of evidence of individual studies was assessed (not outcome specific) |
| Bedretdinova ^9^ | 2020 | 11 | RCTs, NRSs (2403) | Medical treatment, surgical treatment, lifestyle modification | Certainty of evidence of individual studies was assessed (not outcome specific) |
| Dunford ^10^ | 2020 | 40 | NRSs (Not report) | Genital reconstructive surgery | Certainty of evidence of individual studies was assessed (not outcome specific) |
| Rahman ^11^ | 2020 | 25 | NRSs (181018) | Hypertensive | Certainty of evidence of individual studies was assessed (not outcome specific) |

Abbreviations: AUS, artificial urinary sphincter; CM, complete surgical metastasectomy; ^68^Ga-PSMA PET, ^68^Gallium prostate-specific membrane antigen positron emission tomography; NRSs, non-randomized studies; RCTs, randomized controlled trials.

^a^ "inappropriate use of GRADE" refers to evaluate of study quality or the rating of the overall certainty of the body of evidence, rather than the evaluation of the specific outcome.

Supplementary Table S2. Summary of the study characteristics and the number and type of (un)rated outcomes of systematic reviews (n = 49) that rated the outcome-specific certainty of evidence with GRADE

| **Author** | **Year** | **Intervention/**  **Exposure** | **Study design** | **Number of studies** | **Number of participants** | **Number of outcomes rated (Number of unrated outcomes)** | **Number of rated comparisons or study designs** | **Outcomes rated** |
| --- | --- | --- | --- | --- | --- | --- | --- | --- |
| Ravani, P.[18] | 2016 | Active access surveillance and preemptive correction of a newly identified stenosis | RCTs | 14 | 1390 | 8 | 2 (pump blood flow, arterial and venous pressure) | Risk of access loss (1 overall evidence and 2 subgroup evidences), Risk of access thrombosis (1 overall evidence and 2 subgroup evidences), Rate of angiograms, Mortality |
| Badve, S. V.[41] | 2016 | Higher hemoglobin target using ESA | RCTs | 73 | 6732 | 13 | 13 (hemoglobin target, RAAS inhibitor, RAAS active medication, another RAAS inhibitor, Convective hemodialysis therapy, Hemodialysis frequency, Vitamin D compound, Exercise training, Icodextrin, Fluid management on hemodialysis, Lower blood pressure target, Isosorbide mononitrate, Growth hormone) | Change in LVM (13 subgroup evidences) |
| Mustafa, R. A.[12] | 2016 | Lowering the dialysate temperature | RCTs | 26 | 484 | 5 | 1 | Quality of life, Intradialytic hypotension, Symptoms of discomfort, Dialysis adequacy, Change in mean arterial pressure |
| Quach, K., L.[14] | 2016 | Spironolactone or eplerenone | RCTs | 9 | 829 | 2 | 1 | Mortality, hyperkalemia |
| Sinha Ray, A.[39] | 2016 | Treated with intravenous vancomycin | RCTs&NRSs | 13 | 5561 | 1 | NA | Risk of AKI |
| Kelly JT[49] | 2016 | Healthy dietary patterns | NRSs | 7 | 15285 | 1 | 1 | All-cause mortality |
| Köves B[42] | 2017 | Antibiotics | RCTs&NRSs | 50 | 7088 | 21 | 10 (patients without risk factors, patients without risk factors, women with recurrent UTI, in pregnant women (treatment vs placebo/no treatment), pregnant women (single dose vs short term), patients with diabetes mellitus, postmenopausal women, elderly institutionalised patients, patients with renal transplants, prior to urological procedures) | Symptomatic UTI (10 subgroup evidences), Resolution of ABU (6 subgroup evidences), Preterm delivery (2 subgroup evidences), Low birthweight (2 subgroup evidences), Side effects |
| Mallat SG[40] | 2017 | A mammalian target of rapamycin inhibitor-based regimen | RCTs | 28 | 6211 | 20 | 1 | CMV infection (2 subgroup evidences), BK virus infection (2 subgroup evidences), Incidence of other viral (2 subgroup evidences), Composite of incidence of acute cellular or antibody-mediated rejection or DSAs, Graft loss (2 subgroup evidences), Serious adverse events (2 subgroup evidences), Proteinuria (2 subgroup evidences), BK nephropathy defined by kidney biopsy, Wound-healing complications (2 subgroup evidences), eGFR or creatinine clearance, ml/min per 1.73 m2 (2 subgroup evidences), Acute cellular or antibody-mediated rejection, CMV disease |
| Schroeck FR[59] | 2017 | Robot-assisted radical prostatectomy (RARP), intensity-modulated radiotherapy (IMRT), proton beam therapy | NRSs | 49 | NA | NA | 1 | NA |
| Palmer SC[34] | 2017 | IV cyclophosphamide, oral cyclophosphamide, MMF, calcineurin inhibitor, plasma exchange, rituximab, or azathioprine, alone or in combination. | RCTs | 53 | 4222 | 21 | 9 (MMF + calcineurin inhibitor, Calcineurin inhibitor, IV cyclophosphamide + MMF, MMF, Oral cyclophosphamide, Prednisone, Mizoribine, Azathioprine, Plasma exchange) | Complete Remission (7 subgroup evidences), All-cause mortality (7 subgroup evidences), End-Stage Kidney Disease (7 subgroup evidences) |
| Drake T[43] | 2017 | Ureteroscopy | RCTs&NRSs | 47 | 8163 | 3 | 3 (Stone free rate at various time points, Complications, Need for secondary procedure) | Stone free rate at various time points - Stone free at 4 weeks, Complications (Clavien-Dindo Grade 3 only) ≥3, Need for secondary procedure |
| Shim SR[16] | 2016 | Prostatic arterial embolization （PAE） | RCTs&NRSs | 16 | 1047 | NA | NA | NA |
| Pathan SA[38] | 2017 | Nonsteroidal anti-inflammatory drugs | RCTs | 36 | 4887 | 6 | 1 | Pain variance, complete relief, >=50% reduction at 30 min, the requirement of rescue analgesia with NSAID treatment, lower vomiting rates, rescue treatments |
| Sun M[32] | 2018 | Adjuvant vascular endothelial growth factor–targeted therapy | RCTs | 5 | 3693 | 3 | 1 | Time to death from any cause (OS), Time to RCC recurrence (DFS), Grade 3–4 adverse events |
| Valentijn PP[37] | 2018 | Person-centered integrated care | RCTs | 14 | 4693 | 6 | 1 | All-cause mortality, All-cause hospitalization, Health-related quality of life, eGFR, ml/min per 1.73 m2, RRT, Controlled BP,130/80mm Hg |
| Viecelli AK[15] | 2018 | Omega-3 polyunsaturated fatty acid | RCTs | 5 | 833 | 9 | 1 | Primary patency failure, Bleeding, Dialysis suitability failure, Interventions to maintain patency or assist maturation, Access abandonment, GI adverse effects, All-cause mortality, CV mortality, Hospitalization |
| Tikkinen KAO[57] | 2017 | Urological non-cancer surgery | NRSs | 37 | NA | 36 (8) | 4 (Non-fatal symptomatic venous thromboembolism, Fatal venous thromboembolism, Non-fatal bleeding requiring reoperation, Fatal bleeding) | Artificial urinary sphincter (2 subgroup evidences), Prostatectomy and open simple (2 subgroup evidences), Sling surgery for male stress urinary incontinence (2 subgroup evidences), Transurethral resection of the prostate (TURP) or equivalent (4 subgroup evidences), Urethroplasty (2 subgroup evidences), Donor nephrectomy and laparoscopic (4 subgroup evidences), Donor nephrectomy and open (4 subgroup evidences), Recipient nephrectomy, open (4 subgroup evidences), Prolapse surgery (open) (4 subgroup evidences), Reconstructive pelvic surgery (4 subgroup evidences), Percutaneous nephrolithotomy (4 subgroup evidences) |
| Tikkinen KAO[44] | 2017 | Urological cancer surgery | NRSs | 44 | 8521 | 78 (2) | 4 (Non-fatal symptomatic venous thromboembolism, Fatal venous thromboembolism, Non-fatal bleeding requiring reoperation, Fatal bleeding) | Cystectomy (8 subgroup evidences), Nephrectomy (24 subgroup evidences), Nephroureterectomy (4 subgroup evidences), Penectomy (2 subgroup evidences), Primary nerve sparing retroperitoneal lymph node dissection (4 subgroup evidences), Prostatectomy (36 subgroup evidences) |
| Navaneethan SD[19] | 2019 | Metabolic acidosis treatment | NRSs | 14 | 1394 | 4 | 1 | eGFR decline, eGFR decline per year, Progression to ESKD, Urinary ACR |
| Bach KE[54] | 2019 | Healthy dietary patterns | NRSs | 18 | 630108 | 3 (3) | 1 | Incident CKD, eGFR decline, Albuminuria |
| Karavitakis M[29] | 2019 | Alfuzosin 10 mg per day | RCTs&NRSs | 21 | 2958 | 28 (40) | 12 (placebo, tamsulosin 0.4mg, clean intermittent self-catheterization (CISC) before transurethral resection of the prostate, prostatic stent (Urolume), suprapubic catheterization, TURP/enucleation (transvesical or retropubic), Intervention for 7 days, tamsulosin 0.4+alfuzosin 10mg, silodosin 8mg od, no medication, indwelling catheter, bipolar TURP) | Successful TWOC rate (15 subgroup evidences), UR recurrence rate (5 subgroup evidences), Modified Clavien classification system grade (4 subgroup evidences), International Prostate Symptom Score (IPSS), urinary retention rates (2 subgroup evidences), Quality of life score |
| Kim A[28] | 2019 | Retropubic tension-free vaginal tape | RCTs&NRSs | 28 | 2607 | 3 | NA | Objective cure rate, Subjective cure rate, Overall complication |
| Omar MI[60] | 2019 | Empiric medical and nutritional supplements | RCTs&NRSs | 61 | NA | NA | NA | NA |
| Covella B[56] | 2019 | Preeclampsia | NRSs | 21 | 2791732 | 5 | 1 (Preeclampsia) | ESRD, CKD (2 subgroup evidences), Albuminuria, Hospitalization for CKD |
| Kowalewski KF[20] | 2018 | Interrupted suturing for vesicourethral anastomosis | RCTs&NRSs | 9 | 1475 | 6 | 1 (continuous suturing for vesicourethral anastomosis) | Catheterization time, Extravasation rate, Anastomotic time, Hospital stay, Continence at 3, 6, and 12 mo, Development of strictures |
| Donaldson JF[26] | 2019 | TUCL nephroscope | RCTs&NRSs | 25 | 2340 | 65 | 7 (TUCL Laser, TUCL Pneumatic, PCCL, CL, TUCL, Tubeless CL, Traditional CL) | SFR (9 subgroup evidences), Unplanned Procedure (10 subgroup evidences), Major Post-operative Complication (10 subgroup evidences), Late Complication (Urethral Stricture) (10 subgroup evidences), Length of hospital stay (9 subgroup evidences), Duration of Procedure (8 subgroup evidences), Re-Treatment (9 subgroup evidences) |
| Mavrakanas TA[51] | 2019 | Short DAPT in patients with CKD | RCTs | 5 | 16990 | 20 | 1 | Primary composite (2 subgroup evidences), Major bleeding (2 subgroup evidences), Tertiary composite (2 subgroup evidences), All-cause mortality (2 subgroup evidences), CV mortality (2 subgroup evidences), MI (2 subgroup evidences), Stent thrombosis (2 subgroup evidences), Target-lesion revascularization (2 subgroup evidences), Stroke (2 subgroup evidences), Any bleeding (2 subgroup evidences). |
| Zumstein V[13] | 2018 | PAE (Prostatic Artery Embolization) | RCTs&NRSs | 5 | 708 | 7 | 2 (TURP, open prostatectomy) | IPSS, IPSS-QoL, IIEF-5, Maximum urinary flow rate, PVR, Prostate volume, PSA |
| Palmer SC[46] | 2020 | Cinacalcet | RCTs | 36 | 11247 | 45 (10) | 4 (Cinacalcet, etelcalcetid, evocalcet, placebo) | Achievement of PTH (6 subgroup evidences), Hypocalcemia (6 subgroup evidences), Nausea (6 subgroup evidences), Vomiting (6 subgroup evidences), All-cause mortality (6 subgroup evidences), Heart failure (3 subgroup evidences), Serious adverse events (3 subgroup evidences), Fracture (3 subgroup evidences), Serum parathyroid hormone (6 subgroup evidences) |
| Drost FH[47] | 2019 | MRI | NRSs | 43 | 13815 | 8 | 4 (MRI, MRI-targeted biopsy, MRI pathway, Systematic biopsy) | True positives/False negatives (4 subgroup evidences), True negatives/False positives (4 subgroup evidences) |
| Goossen K[21] | 2020 | Icodextrin for peritoneal dialysis | RCTs | 19 | 1693 | 9 | 1 | Mortality, PD technique failure, QoL, Net peritoneal ultrafiltration (3 subgroup evidences), SAE, Pts with peritonitis, Uncontr fluid overload |
| St-Jules DE[30] | 2020 | Phosphate-specific medical nutrition therapy | RCTs&NRSs | 12 | 3119 | 4 | NA | QALY, Serum phosphate levels, Serum phosphate levels, Adverse events |
| Shaman AM[36] | 2020 | ACE inhibitors, ARBs, a-Blockers, b-Blockers, calcium-channel blockers, aldosterone antagonists, renin inhibitors | RCTs | 40 | 4283 | 28 | 1 | Lowering systolic BP (28 subgroup evidences) |
| Berling I[58] | 2020 | Extracorporeal treatment | NRSs | 44 | NA | 6 | 1 | Mortality (2 subgroup evidences), Permanent visual deficit, Length of hospitalization, Serious complications of catheter insertion, Serious complications of ECTR |
| Kallidonis P[27] | 2019 | Combination therapy with α-blocker and PDE5I (Phosphodiesterase-5 Inhibitor) | RCTs | 25 | 2600 | 11 | 5 (IPSS, Qmax, PVR, IIEF, Adverse events) | IPSS at 3 months, IPSS at the end of the follow up, IPSS change, Qmax at the end of the follow up, Qmax change, PVR at the end of the follow up, PVR change, IIEF at the end of the follow up (2 subgroup evidences), IIEF change, Adverse events |
| Abrar M[24] | 2020 | Botulinum toxin A (BTX-A) | NRSs | 17 | 2202 | 9 | 1 | Male gender (3 subgroup evidences), Age (2 subgroup evidences), Urodynamic parameters (3 subgroup evidences), Diabetes |
| Teoh JY[17] | 2020 | En bloc resection of bladder tumour (ERBT) | RCTs&NRSs | 10 | 1155 | 20 | 1 | Operative time (2 subgroup evidences), Irrigation time (2 subgroup evidences), Catheterisation time (2 subgroup evidences), Hospital stay (2 subgroup evidences), Obturator nerve reflex (2 subgroup evidences), Bladder perforation (2 subgroup evidences), Presence of detrusor muscle in specimen (2 subgroup evidences), Recurrence at 0-12 months (2 subgroup evidences), Recurrence at 13-24 months (2 subgroup evidences), Recurrence at 25-36 months (2 subgroup evidences) |
| Pesonen JS[52] | 2019 | Nocturia | NRSs | 9 | 23950 | 4 | 2 (fall, fractures) | Prognosis (2 subgroup evidences), Causation (2 subgroup evidences) |
| Kallidonis P[23] | 2020 | Shock wave lithotripsy (SWL)/Retrograde intrarenal surgery (RIRS) /Percutaneous nephrolithotomy (PCNL) | RCTs | 15 | 2035 | 27 | 3 (PCNL, RIRS, SWL) | Stone free rate up to 3 months (6 subgroup evidences), Operative time (6 subgroup evidences), Hospitalisation time (3 subgroup evidences), Complications (6 subgroup evidences), Secondary procedures (6 subgroup evidences) |
| Pesonen JS[53] | 2019 | Nocturia | NRSs | 11 | 33821 | 2 | 1 | Mortality (2 subgroup evidences) |
| Cai T[25] | 2019 | Fosfomycin trometamol | RCTs | 15 | 2295 | NA | 1 | NA |
| Pilatz A[48] | 2020 | Experimental interventions (including variations and co-interventions) | RCTs | 59 | 14153 | 24 | 11 (no antibiotic prophylaxis, Antibiotic prophylaxis after biopsy, long-term antibiotic prophylaxis, aminogylcosides, cephalosporins, fluoroquinolones, penicillins, sulfonamides, oral antibiotic prophylaxis, Targeted antibiotic prophylaxis, multiple agents for prophylaxis) | Pooled infectious complications (13 subgroup evidences), Hospitalization (11 subgroup evidences) |
| Kim DK[33] | 2020 | PV-UCB (Plasmacytoid Variant Urothelial Carcinoma of the Bladder) | NRSs | 8 | 4004 | 8 | 6 (Pathologic T stage 3 and 4, Lymph node metastasis, Ureteral margin-positive, Perivesical margin-positive, Overall mortality, Cancer-specific mortality) | Pathologic T stage 3 and 4, Lymph node metastasis, Ureteral margin-positive, Perivesical margin-positive, Overall mortality (2 subgroup evidences), Cancer-specific mortality (2 subgroup evidences) |
| Park KJ[35] | 2020 | PI-RADS v2 (The Prostate Imaging Reporting and Data System version 2) | RCTs&NRSs | 13 | 4265 | 4 | 4 (PI-RADS category 1-2, category3, category4, category5) | PI-RADS (4 subgroup evidences) |
| Marchioni M[45] | 2019 | ADT, Apalutamide, Biphosfonate, Celecoxib, Celecoxib plus bisph, Docetaxel, Docetaxel plus bisph, Enzalutamide, bisph | RCTs | 13 | 10800 | 184 (65) | 10 (Abiraterone, ADT, Apalutamide, Biphosfonate, Celecoxib, Celecoxib plus bisph, Docetaxel, Docetaxel plus bisph, Enzalutamide, bisph) | Mortality (80 subgroup evidences), disease progression (23 subgroup evidences), high grade adverse events (81 subgroup evidences) |
| Kelly JT[55] | 2020 | Lifestyle factors, including diet, current smoker, alcohol, physical activity | NRSs | 104 | 2755719 | 24 | 12 (Fish, Fruit, Vegetable, Sugar-sweetened beverage, Carbohydrate, Protein, Phosphate, Potassium, Sodium, Physical activity, Alcohol, Smoking) | Incident CKD (14 subgroup evidences), GFR decline (4 subgroup evidences), Albuminuria (4 subgroup evidences), End-stage kidney disease (2 subgroup evidences) |
| Pyrgidis N[22] | 2020 | Renal transplantation | NRSs | 20 | 1695 | 3 | 3 (After RT, deteriorated EF, postoperative IIEF) | Number of patients with ED before and after RT, Number of patients with improved versus deteriorated EF at postoperative evaluation, Mean difference in preoperative and postoperative IIE F score |
| Sountoulides P[31] | 2020 | Treated with ureteral stent | RCTs&NRSs | 5 | 3309 | 3 | 2 (No ureteral stent, no drainage) | Mortality (3 subgroup evidences) |
| Pradere B[50] | 2020 | Nonantibiotic strategies | RCTs | 90 | 16941 | 14 (6) | 7 (Transrectal prostate biopsy, Extended number of biopsy cores, No periprostatic nerve block, Extended number of injections for periprostatic nerve block, Reusable needle guide, Rectal preparation without enema, Rectal preparation without povidone-iodine) | Pooled infectious complications (7 subgroup evidences), Hospitalization (7 subgroup evidences) |

NRSs: nonrandomized controlled trials; RCTs: randomized trials

Supplementary Table S3. Overview of the GRADE domains of systematic reviews that rated the outcome-specific certainty of evidence

| **Author** | **Year** | **Meta-analysis conducted** | **Strength of recommendations** | **Summary of Findings Table** | **Outcome importance reported** | **Downgrading factors** | **Upgrading** |
| --- | --- | --- | --- | --- | --- | --- | --- |
| Mustafa ^12^ | 2016 | Y | N | Y | Y | RoB (Unclear method for sequence generation and concealment of allocation reported. Blinding, four of ten studies blinded patients, one of ten studies blinded staff, one of ten studies blinded providers, and one of ten studies blinded investigators), Inconsistency (Substantial heterogeneity (I2=63%) that was partially explained by excluding studies with loss to follow-up (I2=18%)) |  |
| Zumstein ^13^ | 2018 | Y | N | Y | N | Not reported |  |
| Quach ^14^ | 2016 | Y | N | N | N | Not reported |  |
| Viecelli ^15^ | 2018 | Y | N | Y | N | Indirectness (data derived from small no. of studies in specific settings that may not be generalizable), Imprecision (risk estimates include null effect and estimate consistent with both appreciable benefit and harm) |  |
| Shim ^16^ | 2016 | Y | N | N | N | Not reported |  |
| Teoh ^17^ | 2020 | Y | N | Y | Y | RoB (Sequence generation and allocation concealment unclear in most studies. Blinding of outcome assessor unclear in most studies. Blinding of outcome assessors unclear in all studies. Allocation concealment unclear in all studies. High risk of bias for allocation concealment in one study and unclear in all others. Potential for confounding bias (see risk of bias graphs) Inconsistency- Important heterogeneity with high I square value. Wide variation in estimates between studies; some studies show benefit for TURBT, others show benefit for EBRT), Imprecision (Wide 95% CI. Very wide 95% CI; contains appreciable benefit and appreciable harm at extremes of the CI. Low event rates contributing to uncertain estimates across studies  d. Wide confidence intervals across studies, many including appreciable benefit and appreciable harm) | Dose-response (strong association) |
| Ravani ^18^ | 2016 | Y | N | Y | N | Not reported |  |
| Navaneethan ^19^ | 2019 | Y | N | Y | N | RoB (Lack of blinding), Indirectness (data derived from small no. of studies), Imprecision (risk estimate includes null effect) |  |
| Kowalewski ^20^ | 2018 | Y | N | Y | N | Not reported |  |
| Goossen ^21^ | 2020 | Y | N | Y | Y | Not reported |  |
| Pyrgidis ^22^ | 2020 | Y | N | Y | Y | RoB (Most studies were of low methodological quality), Inconsistency (High heterogeneity and non-overlapping confidence intervals) | Plausible confounding (all plausible residual confounding would reduce the demonstrated effect), Dose-response (strong association) |
| Kallidonis ^23^ | 2020 | Y | N | Y | Y | RoB (Among the included studies there is high performance bias due to the surgical nature of the intervention that exclude any kind of blinding), Inconsistency (Great heterogeneity detected. There is a statistical heterogeneity among the studies, but the results have one direction. There were different estimates of effect in different studies), Imprecision (The included studies generally have small sample sizes, low event rates and wide confidence intervals) |  |
| Abrar ^24^ | 2020 | Y | N | Y | N | Not reported. |  |
| Cai ^25^ | 2019 | Y | N | N | N | Not reported |  |
| Donaldson ^26^ | 2019 | Y | N | Y | Y | RoB (Singh 2011 did not report any harms data (complications). High attrition rate for late outcomes in Bansal 2016), Inconsistency (I2=84%: some variation in magnitude of effect but consistent direct of effect), Indirectness (unexplained high attrition rate in Bhatia 1994, duration of follow-up not reported in Deswanto 2017), Imprecision (Residual stone fragments likely to be a low incidence event; also narrow CI but this crosses 1.0. no events detected; likely low incidence outcome with small sample size. CI crosses 0, relatively small sample size 4040), Inconsistency (no serious limitation. I2=40%. I 2=52%), Indirectness (variable definition of major bleeding), Imprecision (confidence interval includes no difference. very few events and large confidence intervals) |  |
| Kallidonis ^27^ | 2019 | Y | N | Y | Y | RoB (Most of the RCTs have low Risk of bias), Inconsistency (There were different estimates of effect in different studies), Indirectness (The studies used different PDE5Is with different potential at improving LUTS. They had also different follow-up periods. We solved these problems by sub-categorizing the IPSS score according to the PDE5I that have been used and by creating a forest plot for a follow-up period of 3 months (for IPSS)), Imprecision (The included studies generally have wide confidence intervals) |  |
| Kim ^28^ | 2019 | Y | N | Y | N | RoB (limitation in study design: start with low) | Large effect (upgrade one for large magnitude of effect) |
| Karavitakis ^29^ | 2019 | Y | N | Y | Y | RoB (Refer forest plot containing RoB assessment), Imprecision (95% CI wide and crossed line of no effect) |  |
| St-Jules ^30^ | 2020 | Y | N | Y | N | RoB (Risk of bias from randomization and missing outcomes data. Of the 11 included studies, three had high risk of bias and eight had some concerns in risk of bias using the ROB 2.0 tool. Inconsistency (Heterogeneity of results was moderate I2=63.2%; wide 95% CI. Deviations of intended intervention), Imprecision (Small sample size) |  |
| Sountoulides ^31^ | 2020 | Y | N | Y | Y | RoB (Retrospective design, failure to adequately control confounding), Imprecision (Small number of events) | Plausible confounding (All plausible residual confounding would reduce the demonstrated effect), Dose-response (strong association) |
| Sun ^32^ | 2018 | Y | N | Y | N | Imprecision (Downgraded by one level for imprecision: wide CI that crosses the threshold of a clinically relevant effect) |  |
| Kim ^33^ | 2020 | Y | N | Y | Y | Inconsistency (There was a significant heterogeneity among the studies), Imprecision (The upper and lower limits of 95% CI included both the meaningful benefit and harm) | Dose-response (strong association) |
| Palmer ^34^ | 2017 | Y | N | Y | N | RoB (study limitations (risks of bias including sequence generation, allocation concealment, blinding, attrition from follow-up, selective reporting of outcomes, and other sources of bias), Inconsistency- consistency in treatment effects between studies), Indirectness (directness of the evidence to likely clinical setting, evidence of small study effects (smaller studies with systematically different results from larger studies)), Imprecision (precision of the estimate (imprecision was considered to be present when the confidence interval favored either of the compared treatments)) |  |
| Park ^35^ | 2020 | Y | N | Y | N | Not reported |  |
| Shaman ^36^ | 2020 | Y | N | Y | N | RoB (The majority of evidence was at high or unclear risk of bias), Inconsistency (Confidence intervals for direct and indirect evidence provided different interpretation. Substantial heterogeneity present between the included trials. Only indirect evidence existed, and inconsistency could not be assessed), Imprecision (Confidence intervals for network estimates include values that would favor either treatment) |  |
| Valentijn ^37^ | 2018 | Y | N | Y | N | RoB (One of the studies had an unclear risk of bias on all of the quality domains. Most of the studies had an unclear risk of bias on allocation concealment and/or selective reporting, and high or unclear risk of bias for blinding of participants or outcome assessors), Inconsistency (Wide variance of point estimates across studies and significant heterogeneity between studies. (I2＞50%)), Imprecision (95% confidence interval includes possible benefits from both usual care and integrated care. Adverse event in only small proportion of studies and 95% confidence interval includes possible benefits from both usual care and integrated care) |  |
| Pathan ^38^ | 2017 | Y | N | N | N | Not reported |  |
| Sinha ^39^ | 2016 | Y | N | N | N | Indirectness (The preponderance of evidence was judged to be indirect because 12 of 13 studies compared vancomycin specifically to linezolid) |  |
| Mallat ^40^ | 2017 | Y | N | Y | N | Inconsistency (Heterogeneity detected I2=81%), Imprecision (95% CI does not rule out or confirm difference between the intervention and control groups) |  |
| Badve ^41^ | 2016 | Y | N | Y | N | RoB (No serious limitations: allocation concealment, unclear risk in 3 (33%) trials; blinding of outcome assessors, unclear risk in 2 (22%) trials; incomplete data, unclear risk in 2 (22%) trials; selective outcome reporting, unclear risk in 2 (22%) trials; blinding of participants and investigators, high risk in 8 (89%) trials), Inconsistency (Moderate level heterogeneity (-1); I2=71.21%) |  |
| Köves ^42^ | 2017 | Y | N | Y | Y | RoB (This study is assessed as RoB for blinding of participants and personnel. Most studies have multiple high RoB domains for RoB assessment. Multiple high-risk domains in RoB assessment. The only RCT has multiple high RoB domains for RoB assessment), Inconsistency (Substantial heterogeneity is seen between studies (I2 > 50% or p for chi-square test <0.10),  Imprecision (RR is based on one study only. New published studies might change the pooled effect estimate. The overall sample size is small; the CI cross the clinical decision threshold between recommending and not recommending treatment. Included one observational study and one RCT) |  |
| Drake ^43^ | 2017 | N | N | Y | Y | RoB (Various pre-specified confounders were not balanced or controlled for across the 4 studies. the 6 NRS included are likely to be susceptible to confounding by the pre-specified important confounders as these were either not measured, or not balanced or controlled for across these NRS. The 3 RCTs and 1 QRCT have some risk of bias domains which were 'high' or 'unclear'), Inconsistency (There were different estimates of effect in different studies. Some confidence intervals do not overlap), Imprecision (The included studies generally have small sample sizes, low event rates and wide confidence intervals) |  |
| Tikkinen ^44^ | 2017 | Y | N | N | N | Indirectness (Data for upper bound of certainty interval is based on data for open cystectomy; 2: We considered that the true underlying bleeding rate of zero was implausible for robotic cystectomy. Although the median bleeding estimate was zero, we used the bleeding risk from one study, which, because of study design (multinational) and sample size (n=939) represented a credible alternative. There was only one study reporting on risk of bleeding requiring re-operation (Supplementary Table 4). Risk of bleeding in this study (Stephenson 2004) was 0.00%, which was considered implausible. To estimate the risk we considered the bleeding risk to be half that of open partial nephrectomy. We therefore considered this procedure to have very serious limitations due to indirectness) |  |
| Marchioni ^45^ | 2019 | Y | N | Y | N | Not reported |  |
| Palmer ^46^ | 2020 | Y | N | Y | N | Not reported |  |
| Drost ^47^ | 2019 | Y | N | Y | N | RoB (mainly in the participant selection and reference standard domains) Inconsistency (Considerable, clinically relevant, heterogeneity was observed across pooled study results), Imprecision (Important imprecision was noted, which contributed to decision to downgrade for inconsistency) |  |
| Pilatz ^48^ | 2020 | Y | N | Y | N | RoB (Downgraded by two levels for study design, as most of the studies included in the meta-analysis were judged to be unclear for sequence generation and allocation concealment. Unclear in blinding of outcome), Inconsistency (I2 is almost 55%), Imprecision (Very low numbers of events, wide 95% CI that crosses the null value and the appreciable harm and benefit), Publication bias (Asymmetry on funnel plot especially when it comes to studies with more precise estimate effect favouring single antibiotic agents) |  |
| Kelly ^49^ | 2016 | Y | N | N | N | Not reported |  |
| Pradere ^50^ | 2020 | Y | N | Y | N | RoB (Risk of bias for random sequence generation, blinding, allocation concealment high/unclear in a number of included RCTs), Imprecision (Wide CI (0.05-2.13) and 95% CI includes no effect and wide CI (0.09-1.61)) |  |
| Mavrakanas ^51^ | 2019 | Y | N | Y | N | Not reported |  |
| Pesonen ^52^ | 2019 | Y | N | Y | N | Not reported |  |
| Pesonen ^53^ | 2019 | Y | N | Y | N | Not reported |  |
| Bach ^54^ | 2019 | Y | N | Y | N | RoB (Evidence quality downgraded to low because of the nonrandomized nature of the studies) | Large effect (Evidence certainty upgraded to moderate because of the large association size.) |
| Kelly ^55^ | 2020 | Y | N | Y | N | RoB (Sample population not all general population and not all appropriate confounders adjusted for in primary analyses. Unclear risk of bias across the studies for statistical analysis. High risk of bias for sample population, prognostic indicator and outcomes. High risk of bias across multiple domains), Inconsistency (Confidence intervals overlap. Moderate heterogeneity and confidence intervals overlap. Moderate heterogeneity. High heterogeneity), Indirectness (Outcome assessed in non-standardized manner across the included studies. 2/5 studies conducted in diseased populations), Imprecision (Very wide confidence intervals) |  |
| Covella ^56^ | 2019 | Y | N | Y | N | Not reported |  |
| Tikkinen ^57^ | 2017 | Y | N | N | N | RoB (There were 3 studies reporting on risk of bleeding requiring re-operation (Supplementary Table 9). Risk of bleeding in each of these 3 studies was 0.00%, which was considered implausible. To estimate risk we considered bleeding risk to be half that of laparoscopic donor. We therefore considered this procedure to have very serious limitations due to indirectness), Indirectness (Two studies (Gordon et al. 2005; Kobashi et al. 2003) included patients primarily undergoing sling surgery for stress urinary incontinence whereas 3 studies (Hefni et al. 2006; Moore et al. 2009; Mueller et al; 2014) included patients primarily undergoing vaginal prolapse surgery. Tyson et al 2014 included patients with female sling and vesicovaginal fistular repair. However, all studies included various proportions of patients with concomitant surgeries. Three studies (Descazeaud et al. 2011, Mamoulakis et al. 2011, van Hemelrijck et al. 2013) for VTE but only one study (Mamoulakis et al. 2011) for bleeding included patients undergoing TURP. We rated down for indirectness for bleeding but not for VTE) |  |
| Berling ^58^ | 2020 | N | N | Y | Y | RoB (Patient reports published on effect of ECTR. Uncontrolled and unadjusted for confounders, such as severity of poisoning, coingestions, supportive and standard care, and cointerventions. Confounding by indication is inevitable because ECTR is usually attempted in the sickest patients. Permanent visual deficits varied from field constriction to complete blindness. This outcome was not systematically measured nor reported), Inconsistency (Not rated down for inconsistency because heterogeneity was mainly explained by variation in site of insertion, use of ultrasound, experience of the operator, populations (adults and pediatric), urgency of catheter insertion, practice patterns, and methodologic quality of studies), Indirectness (ECTR and standard care performed may not be generalizable to current practice. Not rated down for indirectness because cannulation and catheter insertion were judged similar to the procedure for other indications), Imprecision (Few events in small sample size: optimal information size criteria not met. Not rated down for imprecision because the wide range reported was explained by inconsistency), Publication bias (Publication bias is strongly suspected due to the study design (patient reports published in toxicology report very severe poisoning either with or without impressive recovery with treatments attempted)) | Plausible confounding (the magnitude of effect is at least expected to be large, which increases the confidence in the estimate of effect) |
| Schroeck ^59^ | 2017 | N | N | N | N | Not reported |  |
| Omar ^60^ | 2019 | Y | N | N | Y | Not reported |  |

1 Wagenaar S, Nederhoed JH, Hoksbergen AWJ, et al. Minimally Invasive, Laparoscopic, and Robotic-assisted Techniques Versus Open Techniques for Kidney Transplant Recipients: A Systematic Review. Eur Urol 2017; 72:205-217

2 Zaid HB, Parker WP, Safdar NS, et al. Outcomes Following Complete Surgical Metastasectomy for Patients with Metastatic Renal Cell Carcinoma: A Systematic Review and Meta-Analysis. J Urol 2017; 197:44-49

3 Han S, Woo S, Kim YJ, et al. Impact of (68)Ga-PSMA PET on the Management of Patients with Prostate Cancer: A Systematic Review and Meta-analysis. Eur Urol 2018; 74:179-190

4 Foroutan F, Friesen EL, Clark KE, et al. Risk Factors for 1-Year Graft Loss After Kidney Transplantation: Systematic Review and Meta-Analysis. Clin J Am Soc Nephrol 2019; 14:1642-1650

5 Campi R, Brookman-May SD, Subiela Henríquez JD, et al. Impact of Metabolic Diseases, Drugs, and Dietary Factors on Prostate Cancer Risk, Recurrence, and Survival: A Systematic Review by the European Association of Urology Section of Oncological Urology. Eur Urol Focus 2019; 5:1029-1057

6 Kotecha P, Sahai A, Malde S. Use of Duloxetine for Postprostatectomy Stress Urinary Incontinence: A Systematic Review. Eur Urol Focus 2020

7 Reus CR, Phé V, Dechartres A, et al. Performance and Safety of the Artificial Urinary Sphincter (AMS 800) for Non-neurogenic Women with Urinary Incontinence Secondary to Intrinsic Sphincter Deficiency: A Systematic Review. Eur Urol Focus 2020; 6:327-338

8 Cacciamani GE, Ghodoussipour S, Mari A, et al. Association between Smoking Exposure, Neoadjuvant Chemotherapy Response and Survival Outcomes following Radical Cystectomy: Systematic Review and Meta-Analysis. J Urol 2020; 204:649-660

9 Bedretdinova D, Ambühl D, Omar MI, et al. What Is the Most Effective Treatment for Nocturia or Nocturnal Incontinence in Adult Women? Eur Urol Focus 2021; 7:453-463

10 Dunford C, Bell K, Rashid T. Genital Reconstructive Surgery in Male to Female Transgender Patients: A Systematic Review of Primary Surgical Techniques, Complication Profiles, and Functional Outcomes from 1950 to Present Day. Eur Urol Focus 2021; 7:464-471

11 Rahman SN, Cao DJ, Monaghan TF, et al. Phenotyping the Association between Nocturia and Hypertension: A Systematic Review and Meta-Analysis. J Urol 2021; 205:1577-1583

12 Mustafa RA, Bdair F, Akl EA, et al. Effect of Lowering the Dialysate Temperature in Chronic Hemodialysis: A Systematic Review and Meta-Analysis. Clin J Am Soc Nephrol 2016; 11:442-457

13 Zumstein V, Betschart P, Vetterlein MW, et al. Prostatic Artery Embolization versus Standard Surgical Treatment for Lower Urinary Tract Symptoms Secondary to Benign Prostatic Hyperplasia: A Systematic Review and Meta-analysis. Eur Urol Focus 2019; 5:1091-1100

14 Quach K, Lvtvyn L, Baigent C, et al. The Safety and Efficacy of Mineralocorticoid Receptor Antagonists in Patients Who Require Dialysis: A Systematic Review and Meta-analysis. Am J Kidney Dis 2016; 68:591-598

15 Viecelli AK, Irish AB, Polkinghorne KR, et al. Omega-3 Polyunsaturated Fatty Acid Supplementation to Prevent Arteriovenous Fistula and Graft Failure: A Systematic Review and Meta-analysis of Randomized Controlled Trials. Am J Kidney Dis 2018; 72:50-61

16 Shim SR, Kanhai KJ, Ko YM, et al. Efficacy and Safety of Prostatic Arterial Embolization: Systematic Review with Meta-Analysis and Meta-Regression. J Urol 2017; 197:465-479

17 Teoh JY, MacLennan S, Chan VW, et al. An International Collaborative Consensus Statement on En Bloc Resection of Bladder Tumour Incorporating Two Systematic Reviews, a Two-round Delphi Survey, and a Consensus Meeting. Eur Urol 2020; 78:546-569

18 Ravani P, Quinn RR, Oliver MJ, et al. Preemptive Correction of Arteriovenous Access Stenosis: A Systematic Review and Meta-analysis of Randomized Controlled Trials. Am J Kidney Dis 2016; 67:446-460

19 Navaneethan SD, Shao J, Buysse J, et al. Effects of Treatment of Metabolic Acidosis in CKD: A Systematic Review and Meta-Analysis. Clin J Am Soc Nephrol 2019; 14:1011-1020

20 Kowalewski KF, Tapking C, Hetjens S, et al. Interrupted versus Continuous Suturing for Vesicourethral Anastomosis During Radical Prostatectomy: A Systematic Review and Meta-analysis. Eur Urol Focus 2019; 5:980-991

21 Goossen K, Becker M, Marshall MR, et al. Icodextrin Versus Glucose Solutions for the Once-Daily Long Dwell in Peritoneal Dialysis: An Enriched Systematic Review and Meta-analysis of Randomized Controlled Trials. Am J Kidney Dis 2020; 75:830-846

22 Pyrgidis N, Mykoniatis I, Sokolakis I, et al. Renal Transplantation Improves Erectile Function in Patients with End-Stage Renal Disease: A Systematic Review and Meta-Analysis. J Urol 2021; 205:1009-1017

23 Kallidonis P, Ntasiotis P, Somani B, et al. Systematic Review and Meta-Analysis Comparing Percutaneous Nephrolithotomy, Retrograde Intrarenal Surgery and Shock Wave Lithotripsy for Lower Pole Renal Stones Less Than 2 cm in Maximum Diameter. J Urol 2020; 204:427-433

24 Abrar M, Pindoria N, Malde S, et al. Predictors of Poor Response and Adverse Events Following Botulinum Toxin A for Refractory Idiopathic Overactive Bladder: A Systematic Review. Eur Urol Focus 2020

25 Cai T, Tamanini I, Tascini C, et al. Fosfomycin Trometamol versus Comparator Antibiotics for the Treatment of Acute Uncomplicated Urinary Tract Infections in Women: A Systematic Review and Meta-Analysis. J Urol 2020; 203:570-578

26 Donaldson JF, Ruhayel Y, Skolarikos A, et al. Treatment of Bladder Stones in Adults and Children: A Systematic Review and Meta-analysis on Behalf of the European Association of Urology Urolithiasis Guideline Panel. Eur Urol 2019; 76:352-367

27 Kallidonis P, Adamou C, Kotsiris D, et al. Combination Therapy with Alpha-blocker and Phosphodiesterase-5 Inhibitor for Improving Lower Urinary Tract Symptoms and Erectile Dysfunction in Comparison with Monotherapy: A Systematic Review and Meta-analysis. Eur Urol Focus 2020; 6:537-558

28 Kim A, Kim MS, Park YJ, et al. Retropubic versus Transobturator Mid Urethral Slings in Patients at High Risk for Recurrent Stress Incontinence: A Systematic Review and Meta-Analysis. J Urol 2019; 202:132-142

29 Karavitakis M, Kyriazis I, Omar MI, et al. Management of Urinary Retention in Patients with Benign Prostatic Obstruction: A Systematic Review and Meta-analysis. Eur Urol 2019; 75:788-798

30 St-Jules DE, Rozga MR, Handu D, et al. Effect of Phosphate-Specific Diet Therapy on Phosphate Levels in Adults Undergoing Maintenance Hemodialysis: A Systematic Review and Meta-Analysis. Clin J Am Soc Nephrol 2020; 16:107-120

31 Sountoulides P, Pyrgidis N, Brookman-May S, et al. Does Ureteral Stenting Increase the Risk of Metachronous Upper Tract Urothelial Carcinoma in Patients with Bladder Tumors? A Systematic Review and Meta-analysis. J Urol 2021; 205:956-966

32 Sun M, Marconi L, Eisen T, et al. Adjuvant Vascular Endothelial Growth Factor-targeted Therapy in Renal Cell Carcinoma: A Systematic Review and Pooled Analysis. Eur Urol 2018; 74:611-620

33 Kim DK, Kim JW, Ro JY, et al. Plasmacytoid Variant Urothelial Carcinoma of the Bladder: A Systematic Review and Meta-Analysis of Clinicopathological Features and Survival Outcomes. J Urol 2020; 204:215-223

34 Palmer SC, Tunnicliffe DJ, Singh-Grewal D, et al. Induction and Maintenance Immunosuppression Treatment of Proliferative Lupus Nephritis: A Network Meta-analysis of Randomized Trials. Am J Kidney Dis 2017; 70:324-336

35 Park KJ, Choi SH, Lee JS, et al. Risk Stratification of Prostate Cancer According to PI-RADS® Version 2 Categories: Meta-Analysis for Prospective Studies. J Urol 2020; 204:1141-1149

36 Shaman AM, Smyth B, Arnott C, et al. Comparative Efficacy and Safety of BP-Lowering Pharmacotherapy in Patients Undergoing Maintenance Dialysis: A Network Meta-Analysis of Randomized, Controlled Trials. Clin J Am Soc Nephrol 2020; 15:1129-1138

37 Valentijn PP, Pereira FA, Ruospo M, et al. Person-Centered Integrated Care for Chronic Kidney Disease: A Systematic Review and Meta-Analysis of Randomized Controlled Trials. Clin J Am Soc Nephrol 2018; 13:375-386

38 Pathan SA, Mitra B, Cameron PA. A Systematic Review and Meta-analysis Comparing the Efficacy of Nonsteroidal Anti-inflammatory Drugs, Opioids, and Paracetamol in the Treatment of Acute Renal Colic. Eur Urol 2018; 73:583-595

39 Sinha Ray A, Haikal A, Hammoud KA, et al. Vancomycin and the Risk of AKI: A Systematic Review and Meta-Analysis. Clin J Am Soc Nephrol 2016; 11:2132-2140

40 Mallat SG, Tanios BY, Itani HS, et al. CMV and BKPyV Infections in Renal Transplant Recipients Receiving an mTOR Inhibitor-Based Regimen Versus a CNI-Based Regimen: A Systematic Review and Meta-Analysis of Randomized, Controlled Trials. Clin J Am Soc Nephrol 2017; 12:1321-1336

41 Badve SV, Palmer SC, Strippoli GFM, et al. The Validity of Left Ventricular Mass as a Surrogate End Point for All-Cause and Cardiovascular Mortality Outcomes in People With CKD: A Systematic Review and Meta-analysis. Am J Kidney Dis 2016; 68:554-563

42 Köves B, Cai T, Veeratterapillay R, et al. Benefits and Harms of Treatment of Asymptomatic Bacteriuria: A Systematic Review and Meta-analysis by the European Association of Urology Urological Infection Guidelines Panel. Eur Urol 2017; 72:865-868

43 Drake T, Grivas N, Dabestani S, et al. What are the Benefits and Harms of Ureteroscopy Compared with Shock-wave Lithotripsy in the Treatment of Upper Ureteral Stones? A Systematic Review. Eur Urol 2017; 72:772-786

44 Tikkinen KAO, Craigie S, Agarwal A, et al. Procedure-specific Risks of Thrombosis and Bleeding in Urological Cancer Surgery: Systematic Review and Meta-analysis. Eur Urol 2018; 73:242-251

45 Marchioni M, Di Nicola M, Primiceri G, et al. New Antiandrogen Compounds Compared to Docetaxel for Metastatic Hormone Sensitive Prostate Cancer: Results from a Network Meta-Analysis. J Urol 2020; 203:751-759

46 Palmer SC, Mavridis D, Johnson DW, et al. Comparative Effectiveness of Calcimimetic Agents for Secondary Hyperparathyroidism in Adults: A Systematic Review and Network Meta-analysis. Am J Kidney Dis 2020; 76:321-330

47 Drost FH, Osses D, Nieboer D, et al. Prostate Magnetic Resonance Imaging, with or Without Magnetic Resonance Imaging-targeted Biopsy, and Systematic Biopsy for Detecting Prostate Cancer: A Cochrane Systematic Review and Meta-analysis. Eur Urol 2020; 77:78-94

48 Pilatz A, Dimitropoulos K, Veeratterapillay R, et al. Antibiotic Prophylaxis for the Prevention of Infectious Complications following Prostate Biopsy: A Systematic Review and Meta-Analysis. J Urol 2020; 204:224-230

49 Kelly JT, Palmer SC, Wai SN, et al. Healthy Dietary Patterns and Risk of Mortality and ESRD in CKD: A Meta-Analysis of Cohort Studies. Clin J Am Soc Nephrol 2017; 12:272-279

50 Pradere B, Veeratterapillay R, Dimitropoulos K, et al. Nonantibiotic Strategies for the Prevention of Infectious Complications following Prostate Biopsy: A Systematic Review and Meta-Analysis. J Urol 2021; 205:653-663

51 Mavrakanas TA, Chatzizisis YS, Gariani K, et al. Duration of Dual Antiplatelet Therapy in Patients with CKD and Drug-Eluting Stents: A Meta-Analysis. Clin J Am Soc Nephrol 2019; 14:810-822

52 Pesonen JS, Vernooij RWM, Cartwright R, et al. The Impact of Nocturia on Falls and Fractures: A Systematic Review and Meta-Analysis. J Urol 2020; 203:674-683

53 Pesonen JS, Cartwright R, Vernooij RWM, et al. The Impact of Nocturia on Mortality: A Systematic Review and Meta-Analysis. J Urol 2020; 203:486-495

54 Bach KE, Kelly JT, Palmer SC, et al. Healthy Dietary Patterns and Incidence of CKD: A Meta-Analysis of Cohort Studies. Clin J Am Soc Nephrol 2019; 14:1441-1449

55 Kelly JT, Su G, Zhang L, et al. Modifiable Lifestyle Factors for Primary Prevention of CKD: A Systematic Review and Meta-Analysis. J Am Soc Nephrol 2021; 32:239-253

56 Covella B, Vinturache AE, Cabiddu G, et al. A systematic review and meta-analysis indicates long-term risk of chronic and end-stage kidney disease after preeclampsia. Kidney Int 2019; 96:711-727

57 Tikkinen KAO, Craigie S, Agarwal A, et al. Procedure-specific Risks of Thrombosis and Bleeding in Urological Non-cancer Surgery: Systematic Review and Meta-analysis. Eur Urol 2018; 73:236-241

58 Berling I, King JD, Shepherd G, et al. Extracorporeal Treatment for Chloroquine, Hydroxychloroquine, and Quinine Poisoning: Systematic Review and Recommendations from the EXTRIP Workgroup. J Am Soc Nephrol 2020; 31:2475-2489

59 Schroeck FR, Jacobs BL, Bhayani SB, et al. Cost of New Technologies in Prostate Cancer Treatment: Systematic Review of Costs and Cost Effectiveness of Robotic-assisted Laparoscopic Prostatectomy, Intensity-modulated Radiotherapy, and Proton Beam Therapy. Eur Urol 2017; 72:712-735

60 Omar MI, Pal RP, Kelly BD, et al. Benefits of Empiric Nutritional and Medical Therapy for Semen Parameters and Pregnancy and Live Birth Rates in Couples with Idiopathic Infertility: A Systematic Review and Meta-analysis. Eur Urol 2019; 75:615-625
